# Behind the scenes of *Popillia japonica* integrated pest management: differentially expressed gene analysis following different control treatments

**DOI:** 10.1186/s12864-025-11949-4

**Published:** 2025-09-01

**Authors:** Claudio Cucini, Rebecca Funari, Giovanni Marturano, Elena Cardaioli, Leonardo Marianelli, Francesco Paoli, Antonio Carapelli, Francesco Frati, Francesco Nardi

**Affiliations:** 1 Department of Life Sciences, University of Siena, Siena, Italy; 2 National Biodiversity Future Center (NBFC), Palermo, Italy; 3 Council for Agricultural Research and Agricultural Economy Analysis (CREA), Florence, Italy

**Keywords:** Japanese beetle, *Metarhizium robertsii*, *Bacillus thuringiensis*, Long last insecticide nets, LLINs, Deltamethrin, IPM, Differential expression analysis, RNA-seq, Gene expression

## Abstract

**Background:**

The Japanese beetle *Popillia japonica* is an invasive pest that is creating a major concern due to its spread and damaging potential. Native to Japan, it was introduced in the U.S.A. and the Azores during the twentieth century, and in mainland Europe in 2014. *P. japonica* is able to attack more than 400 plant species, including some of high economic importance, and the possible losses related to uncontrolled expansion in Europe are substantial. Numerous control measures are being developed to control this pest, with a specific focus on Integrated Pest Management and environmentally safe strategies. Aiming for a genetically informed optimization of the design of these control methods*,* we studied the gene expression response of the pest following exposure to three different treatments: deltamethrin-coated long lasting insecticidal nets, *Bacillus thuringiensis* and *Metarhizium robertsii* spores.

**Results:**

The treatment with insecticidal nets resulted in the differential expression of genes related to Ca^2+^ transport and CYP-based detoxification. Exposure to *B. thuringiensis* was associated with enrichment of gene ontology terms related to antimicrobial peptides and immune function, which suggested potential modulation of immune-related processes. Treatment with *M. robertsii* led to the production of antifungal peptides as well as an up-regulation of the Toll and MAPK pathways.

**Conclusions:**

These findings can be variously interpreted as a response of the insect to minimize the effects of the treatment at the molecular level (e.g. Ca^2+^ increase), as a direct attempt of the insect to combat the agent (e.g. antimicrobial peptides), or as part of a more complex interplay between the insect and the biological control agent (e.g. modulation of the immune system). In general terms, the response to the insecticidal nets and, partly, to *B. thuringiensis*, appears to arise from a direct interaction of the insecticide molecule, or toxin, with their targets at the molecular level. On the other hand, multiple gene pathways are modulated in the response to *M. robertsii*, suggesting a more diversified mode of action that impacts a broader spectrum of biological mechanisms, in line with the notion that the fungus actually grows and reproduces inside the insect host.

**Supplementary Information:**

The online version contains supplementary material available at 10.1186/s12864-025-11949-4.

## Background

*Popillia japonica*, commonly known as the Japanese beetle (JB), is a scarab beetle native to Japan. After an accidental introduction in 1916, *P. japonica* established in North America and, more recently, it managed to spread across Europe (Azores, Italy and Switzerland). The route of invasion has been reconstructed in [1–3], combining historical and distributional data with a variety of molecular markers. The beetle is known for its broad host range and destructive feeding habit as it feeds on the foliage, flowers, and fruits of over 400 plant species, including several economically important crops [4–6]. This generalist feeding habit has been associated with the expansion of specific gene families related to polyphagy [7]. Moreover, due to its prolificacy and high dispersal capability, the beetle is able to spread over long distances via air cargo, posing a threat even to areas that have not yet been invaded [2, 8, 9].

Since the beginning of the last century, several different management strategies have been applied to limit the spread of *P. japonica*, including physical, cultural, chemical and biological control [4, 5]. Physical control entails the use of nets and traps to limit adult beetle spread. Nets are employed to protect plants from a direct attack by adult beetles; traps, which usually contain the female sex pheromone as an attractant, are designed to attract and capture adults*,* thus reducing the local population of the pest. Nevertheless, these strategies have the disadvantage of requiring a substantial manual effort to recover the captured specimens [4]. Cultural control, in turn, includes a wide range of agricultural techniques designed to limit the buildup of populations by limiting the viability of larvae or adults. Examples, exhibiting varying degrees of success, include intercropping with sorghum, that is toxic to the JB, halting irrigation, and weed mulching. A third, widely employed, method to control *P. japonica*’s adults relies on the use of insecticides. This is exemplified by the initial response in the USA post introduction, that involved the massive use of broad-spectrum insecticides in an attempt (albeit failed) to limit its spread. These resulted in unintended adverse effects on non-target species, including birds, pets, and humans [4, 8]. In more recent years, and following the recognition of the significant environmental impact of insecticides, the current European legislation has curtailed the list of approved molecules (https://ec.europa.eu/food/plant/pesticides/eu-pesticides-database/start/screen/active-substances) and, at present, the most promising compounds showing a significant efficacy against adults are acetamiprid, chlorantraniliprole, deltamethrin, etofenprox and λ-cyhalothrin (see [5] for details). In order to limit the undesirable effects of these compounds on non-target species, various methods of application are being considered to replace generalized spraying. These include the use of Long-Lasting Insecticidal Nets with various active ingredients, including deltamethrin, which have been shown to cause 100% mortality in JB adults within 10 days of contact with the net [10].

More recently, with the explicit goal of enhancing environmental sustainability, a variety of biological control measures have been implemented, and are being tested, to limit *P. japonica* populations. These include the use of generalist and specialist predators, the microsporidian *Ovavesicula popilliae* and different species of entomopathogenic nematodes (EPNs; [4, 5]). Yet, the most promising agents of biological control are entomopathogenic bacteria (EPBs) and fungi (EPFs). Among the former, four distinct bacterial species/strains showed high efficacy in infecting and killing *P. japonica* larvae (*Paenibacillus popilliae*, *P. lentimorbus*, and *Bacillus thuringiensis japonensis*) and adults (*B. th.* var. *galleriae*) [5]. The details of the cytotoxic activity of *B. thuringiensis* are still unclear, but the process hinges on crystalline inclusions produced by the bacterium that contain protoxins, such as Cry proteins. Upon ingestion by the insect, these proteins are activated by midgut proteases and bind to receptors on the epithelial cells, leading to the formation of lytic pores in the plasma membranes, the breakdown of the intestinal epithelium, septicemia and death [11]. In turn, while several EPFs were evaluated in recent years (see [5] for an overview), *Metarhizium robertsii* and *M. brunneum* have been demonstrated to be the most effective in killing JB adults. Their efficacy has been tested in laboratory conditions as well as in field trials using a device specifically designed to spread fungal spores in the population [5, 12]. The mechanism of action of *M. robertsii* is similar to that of other *Metarhizium* species: upon host recognition, it forms an appressorium and secretes degradative enzymes to penetrate the insect cuticle as well as antibiotics to evade the insect's cuticular microbiota, leading to a global colonization of the insect hemocoel [13].

Significant advances have been made in recent years on the genetics of *P. japonica,* including genome sequencing [7], the characterization of the expansion of gene families related to detoxification and polyphagy [7], as well as the use of genome-wide SNPs to study the invasion process [3]. Here, we aim at deploying this knowledge for the purposes of Integrated Pest Management (IPM), by optimizing control methods for *P. japonica* on the basis of gene expression information. We therefore studied the defense mechanisms elaborated by the pest as a response to different management conditions using an RNA-seq approach. JB adults from the Italian invasive population were treated using three control agents currently under evaluation: deltamethrin coated long lasting insecticidal nets, the entomopathogenic bacterium *B. th.* var. *galleriae* and spores of the fungus *M. robertsii.* Following RNA-seq, a differential expression analysis (DEA) was conducted to identify differentially expressed genes (DEGs) between two time-points of each treatment and a control group.

## Materials and methods

### Sample collection

Individuals were sampled in the wild in June 2022 in Trecate (Novara, Italy) and acclimated overnight at CREA (Florence, Italy) where they were fed *ad libitum* with common hazel (*Corylus avellana*) leaves. Occasional dead individuals, possibly injured during collection, were removed. After acclimation, samples were randomly divided into four groups of about 70–80 individuals each: a control group (CT) and three treatment groups (long-lasting insecticidal net coated with deltamethrin: LLIN; *B. th.* var. *galleriae:* BTG; *M. robertsii*: MRO – see paragraph 2.2). Individuals from the three treatment groups were collected at two time points following treatment (short and medium exposure) to visualize the progress of the beetle response over time. All experiments were performed under controlled conditions (26 ± 2 °C; 16L:8D photoperiod) in the containment facility at CREA. Following treatment, the collected animals were snap-frozen in liquid nitrogen and stored at −80 °C until molecular analyses.

### Treatment protocols

In line with previous experiments [10, 14], treatment with deltamethrin was carried out using the PermaNet^®^ net with an active ingredient concentration of 4 mg AI/g fiber. Individuals were forced to walk on the net for 90 s, mimicking the exposure within an attract-and-kill device ([15], Fig. S1a). Treated individuals were collected in a single container with a wet cotton as a source of humidity, and fed *ad libitum* with hazel leaves. Insects were sacrificed after 8 h (henceforth LLIN8) and 24 h (henceforth LLIN24).

Treatment with *B. th.* var. *galleriae* was performed in individual Petri dishes (Fig. S1b) where a circular (~ 3.5 cm ⌀) cutting of a common hazel leaf, previously soaked in a liquid culture of *B. th.* var. *galleriae* at a concentration of 23 g/L, was placed on the bottom. Individual housing was required to ensure that all insects were feeding on the contaminated leaf. Insects were kept under conditions as above and sacrificed after 8 h (henceforth BTG8) and 24 h (henceforth BTG24).

Treatment with *M. robertsii* was accomplished using 500 g of rice mixed with spores of the fungus at a concentration of 4.07 × 10^8^/g rice. Insects were forced to walk and roll on the contaminated rice for three minutes, mimicking the exposure in the auto-dissemination device developed by [12] (Fig. S1c). After treatment, samples were collected in a common container and kept as in the LLIN treatment. Individuals were sacrificed after 36 h (henceforth MRO36) and 96 h (henceforth MRO96) from exposure. The prolonged incubation time after exposure, compared to other treatments, was justified by the slower mode of action of the fungus, associated to the necessity of the fungus to develop within the host [10, 12, 16].

### RNA extraction and sequencing

Total RNA for transcriptome sequencing was extracted from the whole body of individual specimens using the QIAGEN RNeasy Micro kit, including a QIAshredder and DNase treatment. Five replicates were prepared for each condition (CT, LLIN8, LLIN24, BTG8, BTG24, MRO36, MRO96; n = 35). mRNA libraries were constructed using the TruSeq Stranded mRNA kit. The 35 libraries were then sequenced with a 150 bp PE layout on a NovaSeq6000 machine at Macrogen (Amsterdam) targeting 9 Giga-bases of sequence output per sample.

### De novo transcriptome assembly and annotation

Raw sequences were quality checked using MultiQC v. 1.17 [17] and trimmed with fastp v. 0.23.2 [18] using default settings. A custom Kraken2 database was assembled in February 2024 and employed to filter reads in Kraken2 v. 2.1.3 [19]. In addition to the standard database (Refseq archea, bacteria, viral, plasmid, human and UniVec_Core), it included plants and fungi to ensure an efficient filtering of contaminating reads from food and from the EPF *M. robertsii*.

To avoid assembly biases from overrepresented sequences and reduce assembly time, while ensuring the largest representation of transcripts, reads from each condition were merged and normalized using BBnorm (available from the BBtools suite: http://sourceforge.net/projects/bbmap). Normalized reads were assembled in Trinity v. 2.1.1 [20]. Following [21], *P. japonica*’s rRNA and mtDNA sequences available in GenBank (NCBI) in March 2024 were downloaded and blasted (blastn v.2.15.0; [22]) against the transcriptome to filter ribosomal and mitochondrial contigs. Very short contigs (< 200nt) were removed. Contigs were finally clustered with cd-hit-est v. 4.8.1 at 95% identity [23]. Transcriptome completeness was evaluated with BUSCO v. 5.5.0 with the endopterygota_odb10 lineage database [24]. Assembly statistics including N50 were calculated with SeqKit stats [25].

Annotation was performed using a custom script [26] based on the Trinotate v. 4.0.0 pipeline (https://github.com/Trinotate/Trinotate/wiki). In brief, transcripts were initially scanned in Transdecoder v. 5.5.0 to predict Open Reading Frames (ORFs). Predicted proteins were searched against the Swissprot database (release 2024_1) using the blast + suite v. 2.15.0 (blastx and blastp; [22]) and the Pfam database (release 36.0) via HMMER v. 3.4 [27]. Transmembrane regions and signal peptide cleavage sites were predicted using tmHMM v. 2.0 [28] and signalP v. 5.0 [29], respectively. A fully complete annotation was obtained with eggNOG (emapper v. 2.1.12; [30]). Finally, functional annotations were summarized in a local SQLite database and merged using Trinotate.

A further refinement of the transcriptome was achieved by eliminating transcripts annotated as non-metazoan based on the taxonomy prediction by eggNOG.

### Differentially expressed genes and gene set enrichment analyses

The assembled transcriptome was used as a reference to quantify non-normalized reads of each treatment group and control (7 groups, 5 replicates, *n* = 35). Transcript quantification was performed using Salmon v. 0.14.1 with the *validateMappings* flag enabled [31]. Read counts were extracted via the *quantmerge* command and used for a differential expression analysis in the DESeq2 v. 1.44.0 R package [32]. DESeq2 read counts were variance-stabilizing transformed (vst) and batch effects removed using *removeBatchEffect* function provided by the limma v. 3.60.2R package [33].

Contrasts were performed for each treatment using the *ashr* shrinkage method [34]: treated-time-point-1 *vs* control; treated-time-point-2 *vs* control; and treated-time-point-2 *vs* treated-time-point-1. Significantly modulated genes were defined as those with adjusted* p*-value (*padj*) < 0.05 and log2 fold change >|2|.

Gene set enrichment analysis (GSEA) for biological processes (BP), cellular components (CC) and molecular functions (MF) was performed separately for each treatment using the R package topGO v. 2.56.0 [35], with the whole transcriptome GO set as background. The *p*-value for terms enrichment was calculated using the Fisher statistic and a weighted algorithm that takes into account the neighboring terms and their relationships.

Data was elaborated and graphically represented in R studio v. 4.4.0 employing ggplot2 v. 3.5.1 [36] and ggVennDiagram v. 1.5.2 [37].

## Results

### Observations on beetle behavior and appearance post-treatment

All insects showed initial symptoms of malaise at the first time point of each treatment and symptoms of generalized illness by the second time point, but very few insects actually died.

In detail, insects exposed to the deltamethrin-baited long lasting insecticide net appeared stunned and were lying on their back after 8 h from the beginning of the experiment, while 24 h after exposure they showed signs of general paralysis, accompanied by the presence of liquid feces. No individual died during the experiment. All insects exposed to *B. th.* var. *galleriae* had eaten a significant portion of the contaminated hazel leaf by the first time point, 8 h after beginning of the experiment. All individuals were characterized by the presence of liquid fecal residues and most appeared dazed, though some managed to move properly. By the second time point, symptoms increased, and affected all individuals alike. No individual died during the experiment. Insects exposed to *M. robertsii* exhibited signs of malaise 36 h after exposure. Ninety-six hours after exposure some insects (~ 23%) were dead (and thus not collected for molecular analyses), while others displayed a fluffy covering on their body surface, suggestive of fungal growth (Tab. S1).

### Transcriptome sequencing, assembly and annotation

RNA sequencing produced an average of 30.1 ± 14.5 million (mean ± standard deviation) reads per sample (Tab. S2). Trimming led to the removal of a maximum of 3.5% of reads and produced a trimmed dataset with quality score > Q35. The initial transcriptome assembly was characterized by high-quality assembly statistics and completeness (94.2% of complete BUSCOs; Tab. S3). Nevertheless, and despite careful filtering before assembly, the functional annotation revealed an excess of non-metazoan transcripts as contaminants (Fig. S2). A second filtering step, at the level of assembled contigs, resulted in a polished version of the assembly, devoid of contaminants. This latter showed a lower transcript count but at no detriment of completeness. The duplication rate was high, as expected in a transcriptome assembly, due to the presence of multiple isoforms per gene (Tab. S3). Most transcripts were successfully annotated in at least one database (Tab. S4), and more than 85% of transcripts were annotated in at least three different databases (Fig. S3).

### Differential expression and gene set enrichment analysis

**Fig. 1:**
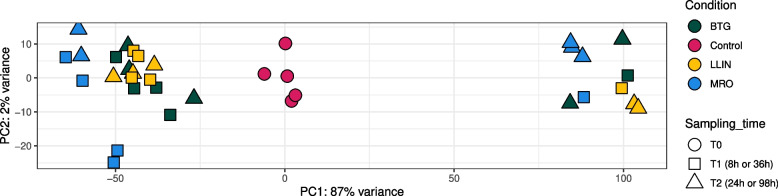
Principal component analysis showing variation in gene expression among treated and control groups. Treatments are color coded whereas different sampling times are represented as shapes

The Principal Coordinate Analysis (PCA) conducted on gene count data produced three distinct clusters, well separated along the first axis (PC1 = 87%; Fig. 1). One corresponded to the control, with all replicates. The other two clusters included all the treated replicates, with no apparent pattern apart for a feeble association with time points (Fig. 1).

**Fig. 2:**
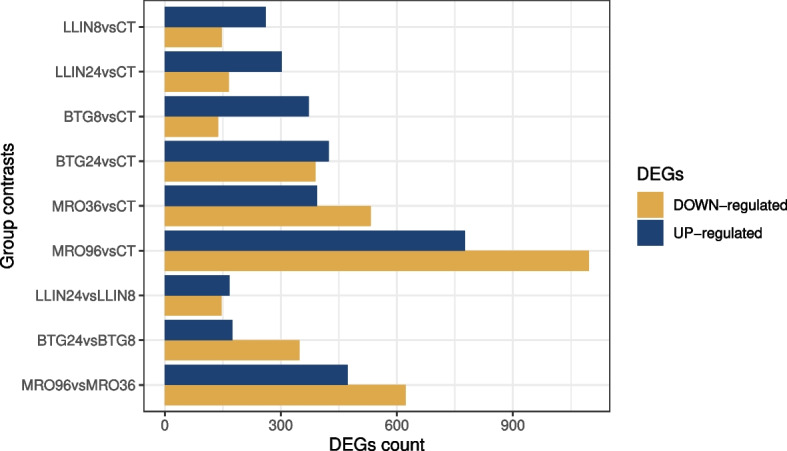
Histogram showing the number of DEG in pairwise contrasts. Up- and down-regulated genes are color coded

A total of 6,928 significantly modulated DEGs (*padj* < 0.05 and log2 fold change >|2|) were observed, with marked differences among contrasts (Fig. 2; Tab. S5). The lowest number of significant DEGs was observed between the two time points of the LLIN treatment (LLIN24*vs*LLIN8, *n* = 313), while the highest was found between the second time point of the *M. roberstii* treatment and control (MRO96*vs*CT, *n* = 1,872) (Fig. 2, displayed as volcano plots in Fig. S4).

**Fig. 3:**
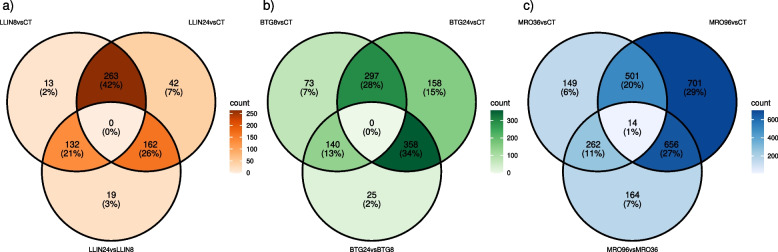
Number and percentage of shared DEGs (*padj* < 0.05) among the two time points of a treatment and control. **a** DEGs shared between the two LLIN treatments and control; **b** DEGs shared between the two BTG treatments and control; **c** DEGs shared between the two MRO treatments and control. Different treatments are shown in different colors, while the color gradient reflects the gene count (the darker the color, the higher the DEG count)

Venn diagrams of DEG sets were used to visualize the number of transcripts co-modulated across contrasts within the same treatment type (Fig. 3). As expected, very few (1%, Fig. 3c) or no transcripts (Fig. 3a,b) were shared among all three contrasts (combinations of control, treated-time-point-1 and 2 of the same treatment). At variance, a substantial fraction of genes (20%−42%) was shared between treated-time-point-1 *vs* control and treated-time-point-2 *vs* control, consistent with these representing two levels of severity (i.e., time since treatment) of the same stress. LLIN and BTG treatments *vs* control, both at the first and second time points, resulted in a small fraction of unique DEGs (2–15%; Fig. 3a,b), whereas MRO96vsCT displayed the highest fraction of unique DEGs (29% at the second time point; Fig. 3c).

Venn diagrams of co-modulated DEG sets across treatment types within the same time point (Fig. S5), in turn, revealed a sizable proportion of shared DEGs among different treatment groups, with 29% overlap at time point 1 and 25% at time point 2 (Fig. S5). The MRO treatment, in turn, resulted in the highest fraction of unique DEGs at both time points (23 and 44%, respectively).

**Fig. 4:**
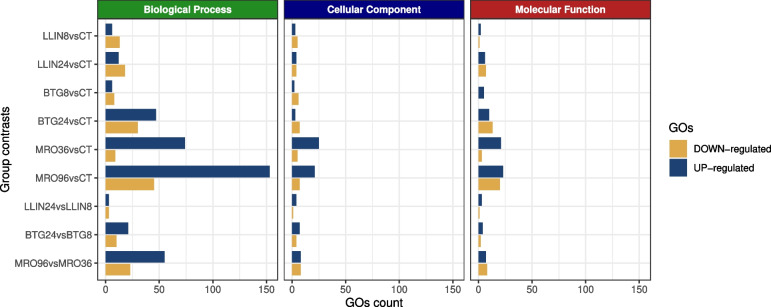
Sharing of modulated GO terms between pairwise contrasts. The three different GO sets (biological process, cellular component and molecular function) are reported in different panels. Up- or down-regulated GOs are color coded

**Fig. 5:**
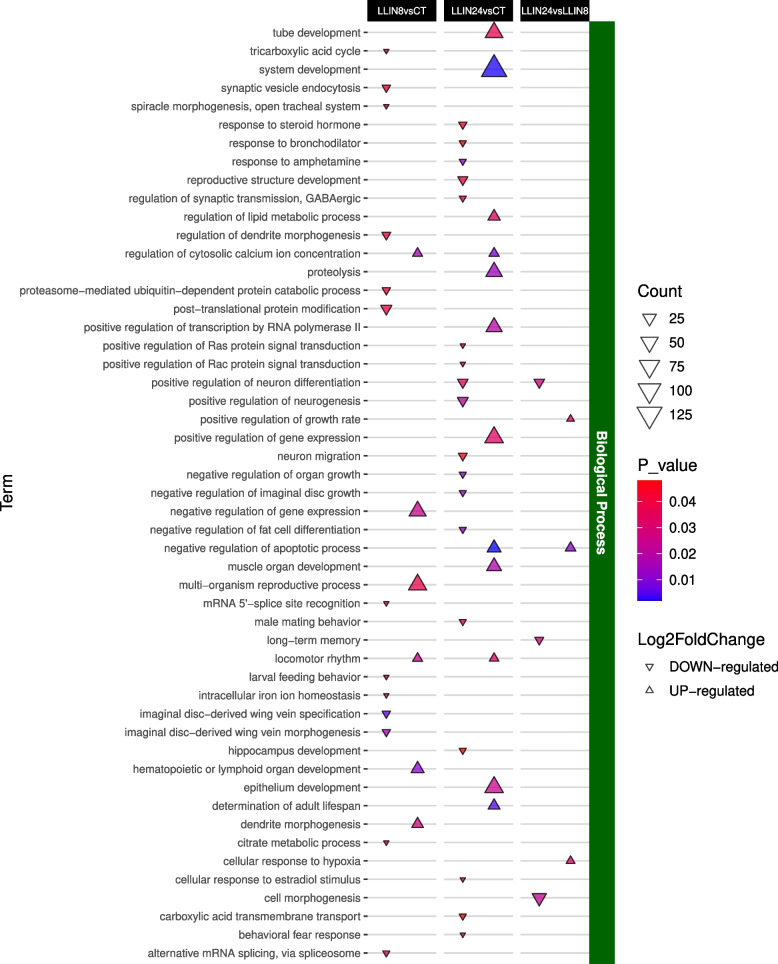
Significant Biological Process GO terms up or down regulated between LLIN treatment comparisons and control. Shapes indicate an up- or down-regulation, their color indicates the *p-value* (< 0.05) and their size reflects the number of modulated GO terms

**Fig. 6:**
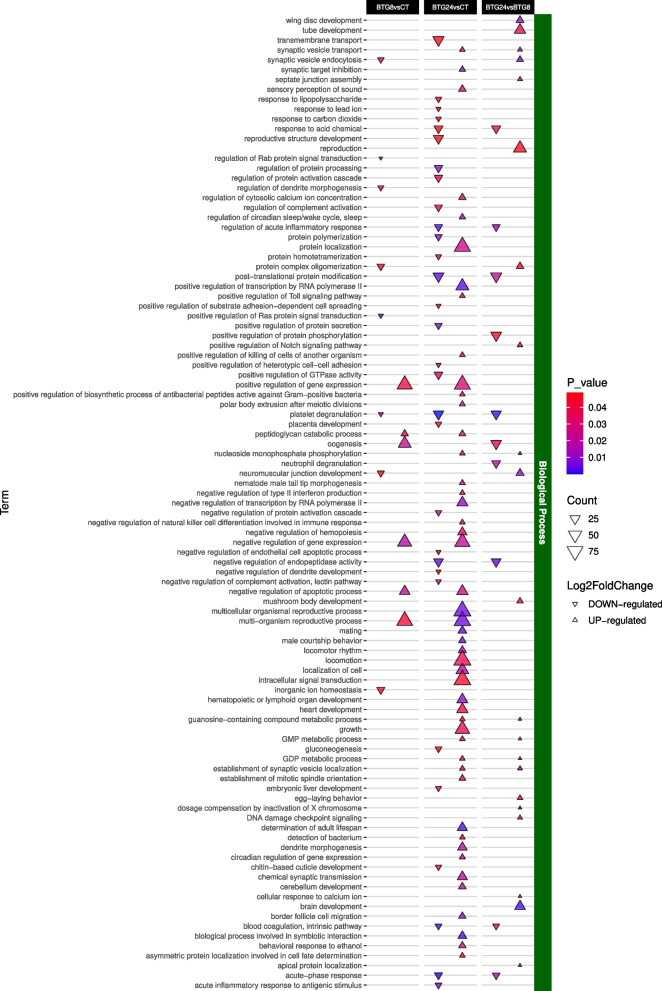
Significant Biological Process GO terms up or down regulated between BTG treatment comparisons and control. Shapes indicate an up- or down-regulation, their color indicates the *p-value* (< 0.05) and their size reflects the number of modulated GO terms

**Fig. 7:**
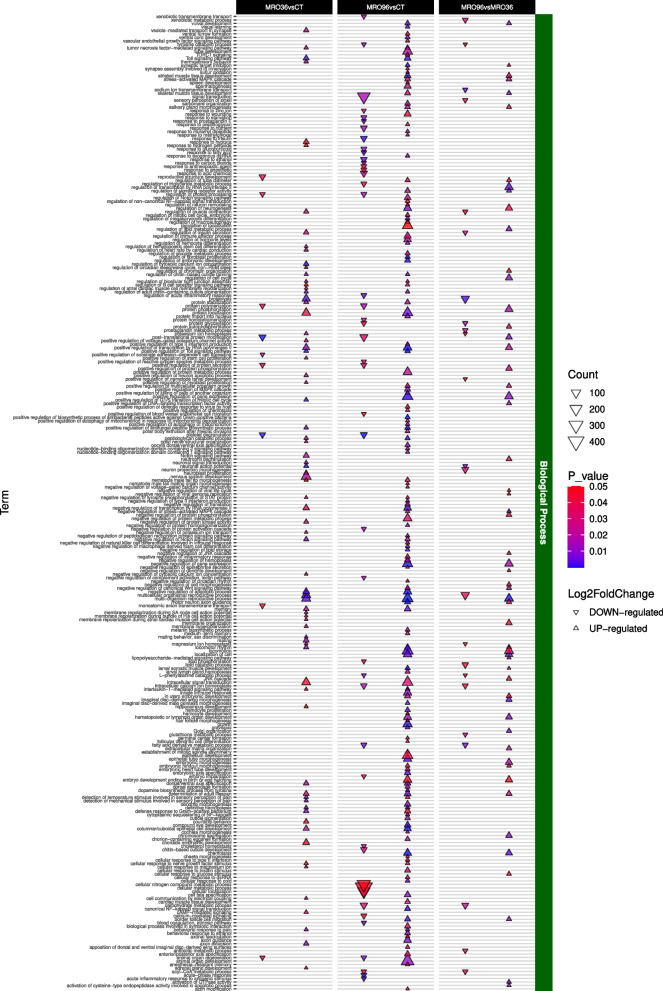
Significant Biological Process GO terms up or down regulated between MRO treatment comparisons and control. Shapes indicate an up- or down-regulation, their color indicates the *p-value* (< 0.05) and their size reflects the number of modulated GO terms

Overall, the GO enrichment analysis identified 795 significant terms (*p*-value < 0.05). Most of these were associated with the MRO96*vs*CT contrast (~ 33%) at the BP level (Fig. 4). While an in-depth analysis of all GO terms was conducted (Figs. 5, 6 and 7, S6-7), the most interesting results were related to the BP (Figs. 5–7).

Transversal to the three treatments, many of the modulated BPs were associated with tissue development. Notably, system development (GO:0048731, 699 DEGs) was enhanced through the up-regulation of multiple transcription factors ‒ for instance, *RUNX1* (mean log2 fold change = 21.86 ± 0.94, due to the expression of multiple isoforms; *padj* = 1.67 × 10^–5^) and *HOXB3* (log2 fold change = 24.70; *padj* = 2.08 × 10^–10^) ‒ in LLIN24*vs*CT. Similarly, striated muscle tissue development (GO:0014706, 92 DEGs) was enriched in MRO96*vs*CT, driven by the up-regulation of *VGLL2* (log2 fold change = 2.5; *padj* = 2.0 × 10^–2^). Additionally, all second time point contrasts *plus* MTO36*vs*CT showed an inhibition of the apoptotic process (GO:0043066, 226 DEGs notably driven by *TSC22D2* (log2 fold change = 5.33; *padj* = 1.9 × 10^–3^). A term designed for adult lifespan (GO:0008340, 144 DEGs) was also modulated.

The LLIN treatment resulted, at both time points, in a marked activation of the regulation of cytosolic calcium ion concentration (GO:0051480, 61 DEGs). For example, *CAC1G* showed a log2 fold change of 21.35 and *padj* of 1.24 × 10^–5^ (Fig. 5). Furthermore, this treatment led to significant up-regulation of cytochrome P450 genes — including *CYP4V2* (log2 fold change = 7.99; *padj* = 4.71 × 10^–5^) and *CYP6a9* (log2 fold change = 5.13; *padj* = 5.78 × 10^–5^)— as shown in Fig. S4 and Tab. S5.

The BTG treatment resulted in few gene ontologies significantly modulated at the first time point and a higher number at the second time point (Fig. 6). Among them were terms related to the presence of the bacterium (GO:0016045, 19 DEGs), antibacterial peptides against Gram-positive bacteria (GO:0006965 25 DEGs), and immune system suppression (GO:0032827, 19 DEGs). All the three terms were up-regulated due to the over-expression of *PGLYRP1/3* (log2 fold change = 5.64; *padj* = 1.76 × 10^–7^). Additionally, the GO term GO:0044403 (involvement in symbiotic interactions) was up-regulated at the second time point in both the MRO and BTG treatments (MRO96*vs*CT and BTG24*vs*CT, 82 and 71 DEGs respectively; Figs. 6 and 7, Table S5).

Treatment with the EPF *M. robertsii* revealed several common terms across both the first and second time points when compared to the control group (MRO36*vs*CT and MRO96*vs*CT; Fig. 7). Specifically, the Toll signaling pathway was positively regulated in treated samples (GO:0005121, GO:0008063, GO:0045752; Figs. 7, S6) involving 86 DEGs including *spätzle* (mean log2 fold change = 4.4 ± 2.16, averaged across expressed isoforms; *padj* = 1.9 × 10^–3^). Terms related to cuticle pigmentation (GO:0007564, GO:0048067, GO:0048082, 37 DEGs were) were also up-regulated, exemplified by the overexpression of *yellow-g2* (log2 fold change = 4.71; *padj* = 1.1 × 10^–3^). The treatment further elicited transcriptional responses associated with inflammation, sensory perception, and behavior. In particular, genes linked to interleukin-1–mediated inflammation (GO:0070498, 26 DEGs) were up-regulated due to the modulation of *NFKB1* (log2 fold change = 11.70; *padj* = 4.79 × 10^–11^). Similarly, the GO term thermosensory behavior (GO:0040040, 28 DEGs) was enriched due to the up-regulation of *TRPA1* (log2 fold change = 2.94; *padj* = 2.74 × 10^–5^). Enhanced expression of *CRHR2* (log2 fold change = 36.05; *padj* = 1.53 × 10^–11^) supported the activation of several GO terms related to pain-related processes (GO:0048266, GO:0050965, GO:0050966, 66 DEGs). The response also involved the antifungal peptide synthesis (GO:0006967, 39 DEGs) again associated with *spätzle* up-regulation. Moreover, locomotion (GO:0040011, GO:0045475, 495 DEGs) was significantly enhanced, underpinned by the up-regulation of several genes involved in skeletal muscle function, including *KCNMA1*, which showed a remarkably high log2 fold change of 38.43 ± 0.76 (*padj* = 2.42 × 10^–11^). Among the GO terms up-regulated at the second time point (MRO96*vs*CT), several were associated with immune responses. Although conflicting GOs related to both negative and positive regulation of the MAPK cascade were identified (GO:0032873, GO:0043410, GO:0051403, 123 DEGs in total), haemocytes were observed to undergo development, differentiation, and proliferation (GO:0007516, GO:0035172, GO:0045610, 15 DEGs) primarily driven by the up-regulation of multiple *pvr* isoforms (log2 fold change = 3.99 ± 0.91; *padj* = 1.5 × 10^–3^). Interestingly, various terms were also linked to the presence of viral elements (GO:0043330, GO:0071359, GO:0002230, 65 DEGs). This response was characterized by the significant over-expression of *dredd*, a key gene in antiviral defense (log2 fold change = 2.92; *padj* = 9.8 × 10^–4^).

## Discussion

The scientific literature on *P. japonica* has burgeoned in recent years, leading to important insights into its ecology, dispersal, and population dynamics [2, 3, 8, 9]. Likewise, different management strategies have been developed to control this invasive species and are being evaluated in field and semi-field tests [4, 10, 15]. Among these methods, several have shown a significant impact on reducing adult beetle populations. To better understand the beetle’s transcriptional response to common pest management strategies currently under development, we studied the modifications in gene expression in *P. japonica* adults after exposure to deltamethrin-coated long lasting nets, *B. th.* var. *galleriae*, and the EPF *M. robertsii.*

At variance with previous studies focusing on the efficacy of such methods, which generally assessed the viability of beetles over medium/long periods following exposure (i.e., end mortality), we investigated the beetles'response over short/medium-term periods to capture the active transcriptomic response of the insect to the stress posed by the exposure to a control mechanisms, while avoiding background noise related to the global collapse of molecular mechanisms and approaching death. The observation that all treated insects showed growing levels of malaise through the experiment, but few insects actually died, confirmed the appropriateness of both sampling points, that apparently cover the full range of the treatment effects. Noteworthy, this different experimental setup does not allow to compare mortality statistics with previous accounts, as those are generally based on 3x  to 9x  longer exposure times. For instance [16], observed a significant mortality three days after exposure to a similar formulation of *B. th.* var. *galleriae*, whereas no dead beetles were observed in our experiments at 8 and 24 h post exposure. Similarly, [38] observed 100% mortality of *P. japonica* larvae after 14 days post exposure to *M. robertsii*, whereas only a few deaths were observed here after 96 h. On the other hand, the results from the LLIN treatment can be readily compared to those of [10], where the authors conducted a time-tracked exposure. Consistent with their findings, we similarly observed that almost all specimens were paralyzed 24 h after exposure to deltamethrin.

From a purely technical standpoint, our results underline the importance of a careful filtering of the transcriptome assembly, something that was not unexpected given the complexity of the system under study (i.e., *P. japonica plus* vegetal material as food *plus* the biological control agent; Tab. S3). Despite the construction of a custom database of contaminants, the first filtering performed at the level of sequence reads was not sufficient. A second round of filtering performed at the assembled-transcripts level was therefore necessary to eliminate contaminant sequences. This is in line with the idea that Kraken2 assigns sequence taxonomy based on a *k*-mer approach on short reads, whereas the functional annotation assigns taxonomy based on database matching on the full transcript.

### Long lasting insecticidal net (deltamethrin) treatment

The use of pyrethroids to control pest populations is a common method employed across a wide range of species. Among this class of compounds, deltamethrin is one of the most effective commercial insecticides [39]. From a biological point of view, deltamethrin targets the nervous system by delaying the closure of voltage-sensitive Na^+^ channels (as first target sites) or Cl^−^/Ca^2+^ channels (as secondary target sites) leading to tremors, paralysis and death of the insect [39]. Contrary to expectations, the DEGs analysis of the LLIN treatment group did not result in a clear indication of paralysis or reduced locomotory processes, nor a reduction of the activity of sodium channels. However, a marked up-regulation was observed of genes associated with cytosolic calcium concentration as the voltage-senstive calcium channel *CAC1G*. This suggests that, following exposure to deltamethrin, the beetles may experience an imbalance in Ca^2^⁺ concentration, which is a key component of the insecticide's mode of action. In response, the insect may be transcriptionally activating enzymes involved in the regulation of cytosolic calcium levels in an effort to mitigate the effects of the insecticide and restore physiological Ca^2^⁺ concentrations (Fig. 5). Alongside Ca^2+^ channels, a modulation was observed in CYP enzymes, that are known in insects to have a defensive role through the detoxification of xenobiotics, including insecticides and deltamethrin (Ravula and Yenugu 2021). The up-regulation of *CYP6a9* (at both time points) and *CYP4V2* (at the second time point) following deltamethrin exposure suggests that the insect may be selectively overexpressing specific cytochrome P450 (CYP) genes to metabolize the insecticide, thereby mitigating its effects. Adding to their significance, both these genes belong to CYP clans that were shown to have undergone substantial gene amplification in the *P. japonica* genome and have been therefore identified as relevant contributors to the molecular arsenal of the species [7]. Incidentally, CYP up-regulation at large was not exclusive of the LLIN treatment and could therefore be interpreted as part of a more generalized response of the insect to xenobiotics, including those of biological origin produced by EPBs and EPFs.

### Bacillus thuringiensis var. galleriae treatment

*B. thuringiensis* comprises several strains that are commonly employed as biological control agents against invasive and pest species. The entomopathogenic action of *B. thuringiensis* takes place through the production of Cry proteins that, activated by proteases that are present in the insect midgut, bind to receptors in the intestinal cells, giving rise to pores that ultimately lead to septicemia and the insect death [11]. Under sublethal conditions, the insect can counteract the bacterial infection through innate immunity mechanisms, such as phagocytosis, or the production of antimicrobial peptides [11]. In our experiment, we did find evidence for a generalized response to bacteria and, specifically, for the production of antibacterial peptides against Gram-positive bacteria (i.e., the bacterial category that includes *B. thuringiensis*). This can be readily interpreted as an attempt of the insect to combat *B. thuringiensis* that is liable to be recognized as an external agent by the immune system and trigger a response, although it may not be the most immediate source of harm to the insect, as crystal inclusions of Cry proteins are produced by the EPB before it gets into contact with the insect. Assessing bacterial load at multiple time points may help clarify whether the observed immune response is directly attributable to *B. thuringiensis* infection. At variance, no evidence emerged of phagocytosis-related processes suggesting that the insect privileged its humoral innate immune system over the cellular innate defense mechanisms to fight the bacterial infection through the production of Peptidoglycan Recognition Proteins (PGRPs) PGLYRP1/3 as also demonstrated in *Drosophila* [40]. Interestingly, and perhaps counterintuitively, a partial suppression of the insect’s immune system was observed during the medium-term treatment (BTG24). Unexpected as it is, this event has been already observed in the lepidopteran *Plutella xylostella* which, upon infection with *B. thuringiensis,* experiences a general down-regulation of its immune system [41]. Highlighting the intricacy of this *pas de deux*, this down-regulation could be hypothesized to be the result of a direct action of the bacterium to circumvent the host defenses during infection.

### Metarhizium robertsii treatment

In 2023, *M. robertsii,* a worldwide distributed species of EPF, was identified in the soil of the Ticino Valley. Subsequent trials on *P. japonica* resulted in a very high mortality rate in adult beetles, making it one of the most promising biological control agent for this pest [38]. Its mode of action encompasses host recognition, the formation of an *appressorium*, cuticle penetration and fungal growth within the insect hemocoel, eventually leading to the insect’s death [13]. Compared to the other two treatments studied, the infection with *M. robertsii* induced the modulation of a higher number of genes and GO terms, suggesting a higher complexity of the host response during infection. This complex and phased host response likely reflects the slower, progressive nature of fungal pathogenesis, where mortality results from gradual tissue invasion and systemic colonization, rather than acute toxicity. Accordingly, the observed delay in mortality (96 h) may stem from the time required for the fungus to penetrate the cuticle, overcome immune defenses, and reach lethal thresholds in the hemocoel—processes that are mirrored by the sustained and evolving gene expression profile observed across time points. Among the observed modulations, the production of antifungal peptides, driven by the positive modulation of *spätzle*, may represent a direct attempt by *P. japonica* to counteract the fungal infection. At the molecular level, the enrichment analysis identified an up-regulation of Toll and cuticle-related terms possibly associated with the ecdysone pathway. This was evident at both time points, suggesting that these pathways remain active during all phases of infection. A similar response has already been described in locusts, where the Toll pathway is activated in fat bodies and haemocytes in response to the infection [42] and it contributes to: (i) the production, in conjunction with the ecdysone pathway, of antifungal peptides; (ii) the triggering of behavioral fever to slow down the infection [43]; and (iii) haemocyte differentiation. Conversely, MAPKs, likely important to promote haemocyte differentiation, appeared to be activated only at the second time point, in line with previous results on other insect species [44]. The modulation of terms related to pain and inflammation linked to interleukin-1, and strictly related to the over-expression of *NFKB1*, was also observed as a possible by-product of the ongoing infection. In summary, the response elicited by an infection with *M. robertsii* appears to be more complex and multifaceted than the responses to LLIN and BTG exposure. It includes a direct attempt to combat the fungus, mediated by the production of antifungal peptides, as well as a longer-term response at the cellular level. The observed complexity in the response to the entomopathogenic fungus is not unexpected for two primary reasons. On one hand, the fungus gradually proliferates to invade the insect hemocoel, establishing prolonged and intimate contact that is likely to stimulate diverse immune responses in the insect. On the other hand, *M. robertsii*, along with other closely related EPF species, are likely common and persistent parasitic symbionts of *P. japonica*. As such, the insect may have evolved strategies to mitigate the damage caused by the infection and coexist with the fungus over the long term.

A possible involvement of viral elements associated to the *M. robertsii* infection, while here supported only by the observed modulation of a term associated to the presence of viral elements, should be investigated in more detail. While still a partially unexplored field, the presence of mycoviruses, as well as their possible role as contributors to the entomopathogenic significance of the fungus, has been studied in the related *Beauveria bassiana* [45]. More recently, transfection of natural mycoviruses into commercial strains of *Metharhizium* was used to enhance the virulence of the fungus in control actions against lepidopteran pests [46]. This suggest the opportunity to characterize mycoviruses that may be present in *M. robertsii* in sight of their potential use as synergistic agents and enhancers of entomopathogenicity.

## Conclusions

In order to optimize innovative control methods for *P. japonica* based on gene expression information, some conclusions can be drawn from the results presented here. One key conclusion concerns the complexity of the biological action, assessed by the number of differentially expressed genes and the biological spectrum (GO terms) of modulated genes. Compared to long-lasting insecticidal nets and *B. th.* var. *galleriae*, the treatment with *M. robertsii* resulted in a substantially larger number of DEGs, as well as a broader range of modulated GOs. This implies a more diversified mode of action, impacting a wider spectrum of biological mechanisms. Notably, this supports the idea that *M. robertsii* is capable of exerting a broader and longer-term control action on the insect.

Furthermore, and most importantly, this observation is significant for understanding the insect's potential to develop resistance to control measures — a common, often rapid and powerful process in insects. A complex and multifaceted mode of action implies that multiple biological processes in the insect are targeted, meaning multiple processes may need to evolve in response to selective pressure to develop resistance. In contrast, a simple mode of action, involving a single interaction between an insecticide and its molecular target, can more easily lead to resistance through as little as a single-point mutation that prevents insecticide binding.

A second aspect is the nature of the response, which is related to the nature of the threat. The treatment with long-lasting insecticidal nets triggers a response involving increased calcium concentration and overexpression of detoxifying enzymes — a fast and specific molecular-level reaction. In contrast, treatments with *M. robertsii* and *B. thuringiensis* var. *galleriae* activate the insect’s immune response, primarily humoral immunity, as well as other functions generally associated with infections by biological entities (bacteria or fungi), resulting in a more complex and gradual response. Therefore, the response to long-lasting insecticidal nets is likely fast and all-or-nothing, whereas the response to *M. robertsii* and *B. thuringiensis* var. *galleriae* unfolds over the medium term and may develop into a dynamic competition (and, possibly, coevolution) between the insect and the biological control agent.

In evaluating control strategies for *P. japonica*, it is important to consider not only the transcriptomic response but also factors like efficacy, cost, environmental safety, and practical deployment. Given that deltamethrin's mode of action targets a limited number of specific sites, resistance could potentially arise through adaptations or point mutations in those targets. This makes long-lasting insecticidal nets more suitable for localized, high-value-crop protection where immediate impact is crucial, while *B. th.* var. *galleriae* and especially *M. robertsii* — with their complex, multifaceted modes of action — may be more appropriate for long-term containment across larger areas, where sustained population control is the priority.

## Supplementary Information

Supplementary Material 1.

Supplementary Material 2.

## Data Availability

All sequence data are available from the Short Read Archive (SRA) under PRJNA860365.

## Abbreviations

JB: Japanese Beetle
IPM: Integrated Pest Management
EPF: Entomopathogenic fungi
EPN: Entomopathogenic nematodes
AI: Active ingredient
DEG: Differential expressed genes
DEA: Differential expression analysis
GO: Gene Ontology
BP: Biological Process
MF: Molecular Function
CC: Cellular Component
