# Supplementary material for: Behind the scenes of Popillia japonica integrated pest management: differentially expressed gene analysis following different control treatments
Source: BMC Genomics. 2025 Sep 1;26:788. doi: 10.1186/s12864-025-11949-4 (PMC12400702; doi:10.1186/s12864-025-11949-4)
Supplement: Supplementary file 1 — Supplementary Material 1. [file 12864_2025_11949_MOESM1_ESM.zip › FigS1.pdf]

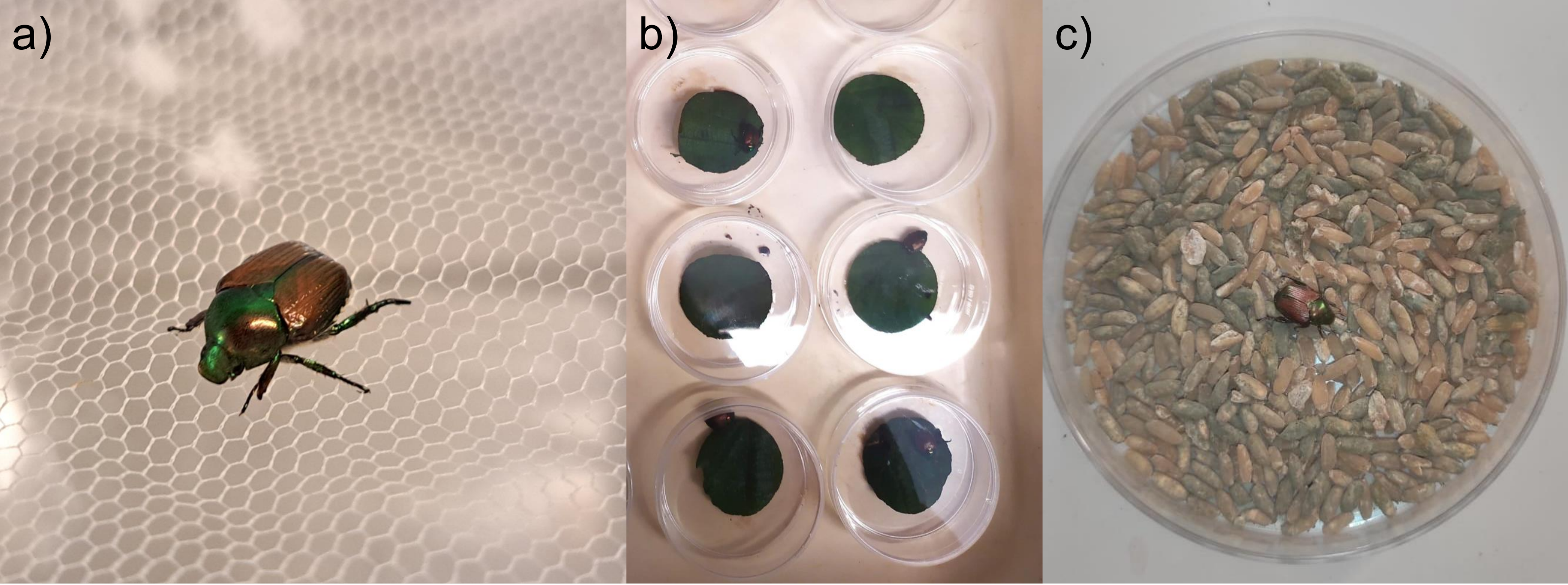

Supplementary Figure S1. Treatment of *P. japonica* adults. Panel a) a JB adult forced to walk on a long lasting insecticidal net coated with deltamethrin for 90 seconds (photo taken using a different net, for illustrative purposes only). Panel b) treatment with *B. th.* var. *galleriae.* Adults of *P. japonica* were fed with a portion of common hazel leaf contaminated with the bacterium in individual petri dishes for the entire length of the experiment. Panel c) treatment with *M. robertsii*. Insects were forced to walk and roll on rice contaminated with fungal spores for three minutes.
