# Supplementary material for: Behind the scenes of Popillia japonica integrated pest management: differentially expressed gene analysis following different control treatments
Source: BMC Genomics. 2025 Sep 1;26:788. doi: 10.1186/s12864-025-11949-4 (PMC12400702; doi:10.1186/s12864-025-11949-4)
Supplement: Supplementary file 1 — Supplementary Material 1. [file 12864_2025_11949_MOESM1_ESM.zip › FigS2.pdf]

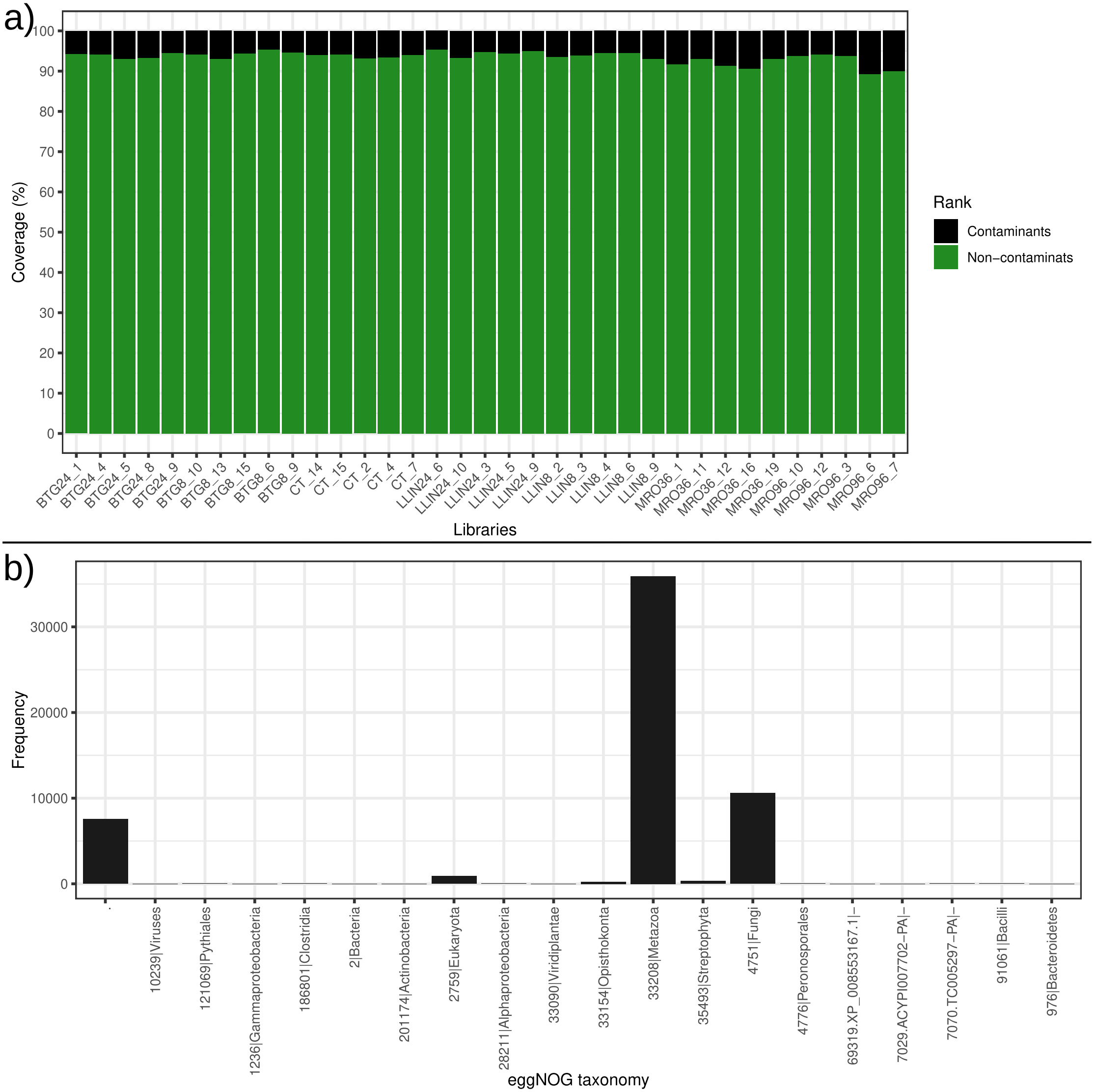

Supplementary Figure S2. Frequency of contaminant reads at different steps of the transcriptome analysis. Panel a) Kraken2 contamination report on raw read data. Contamination percentages range between 4.60% (BTG8_6) to 10.72% (library MRO96_6). Library names refer to the treatment group (BTG: exposure to *B. th.* var. *galleriae*, CT: control group, LLIN: exposure to deltamethrin, MRO: exposure to *M. robertsii*) and the replicate number. Panel b) taxonomic attribution of transcripts (before filtering) according to eggNOG taxonomy. The first bar, identified with a dot, represents transcripts with no taxonomic identification.
