# Supplementary material for: Behind the scenes of Popillia japonica integrated pest management: differentially expressed gene analysis following different control treatments
Source: BMC Genomics. 2025 Sep 1;26:788. doi: 10.1186/s12864-025-11949-4 (PMC12400702; doi:10.1186/s12864-025-11949-4)
Supplement: Supplementary file 1 — Supplementary Material 1. [file 12864_2025_11949_MOESM1_ESM.zip › FigS4.pdf]

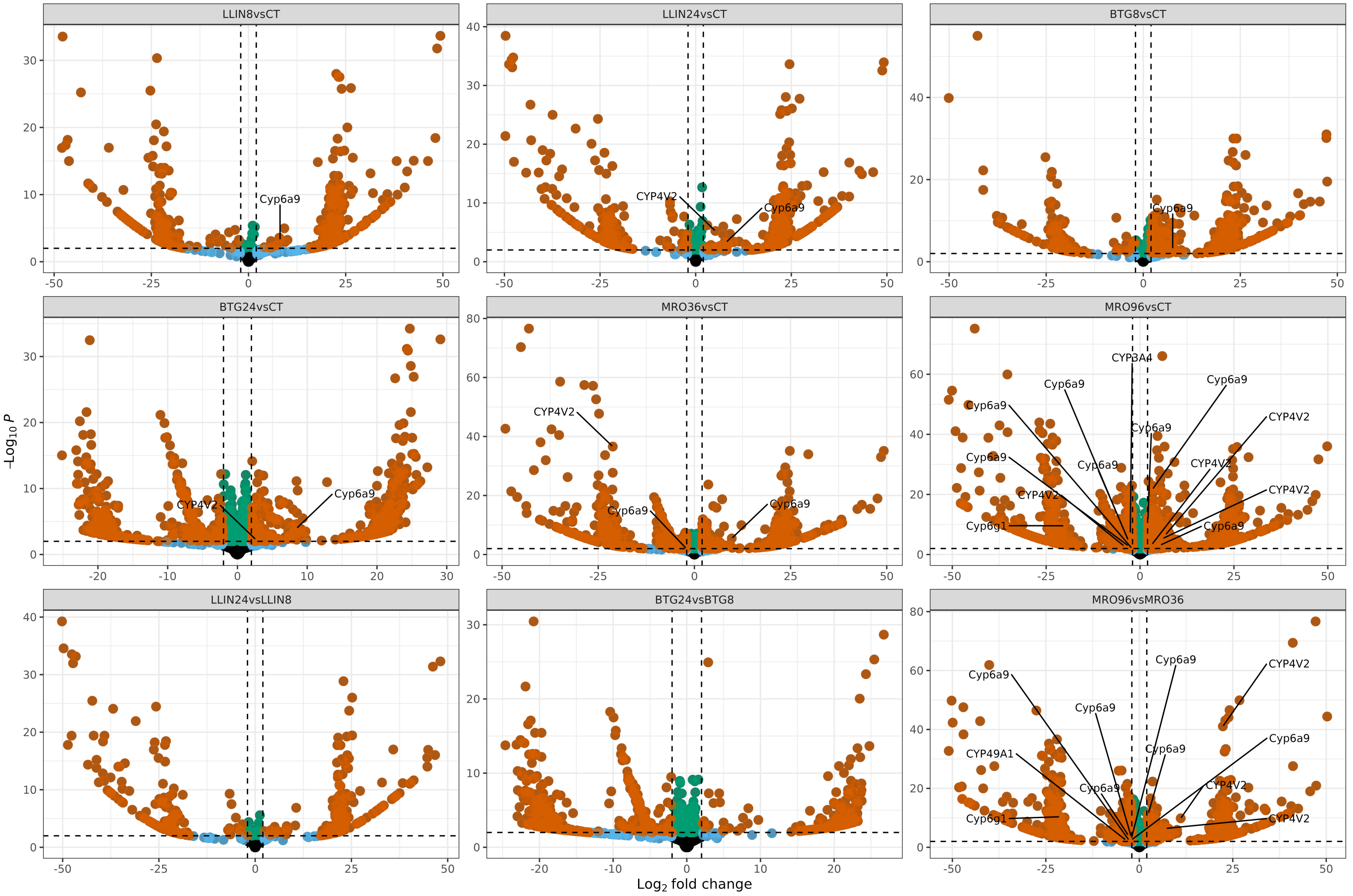

Supplementary Figure S4. Volcano plot visualization of DEGs. The Y and X axes represent the *padj* (as -log10) and the log2 fold change, respectively. DEGs are depicted as dots, with thresholds for significance set according to the criteria detailed in the Materials and Methods section: log2 fold change > |2| and *padj* < 0.05. Colors are used to distinguish DEGs that meet these thresholds, with significant and abundant DEGs highlighted in orange. *CYP* genes are indicated.
