# Supplementary material for: Behind the scenes of Popillia japonica integrated pest management: differentially expressed gene analysis following different control treatments
Source: BMC Genomics. 2025 Sep 1;26:788. doi: 10.1186/s12864-025-11949-4 (PMC12400702; doi:10.1186/s12864-025-11949-4)
Supplement: Supplementary file 1 — Supplementary Material 1. [file 12864_2025_11949_MOESM1_ESM.zip › FigS5.pdf]

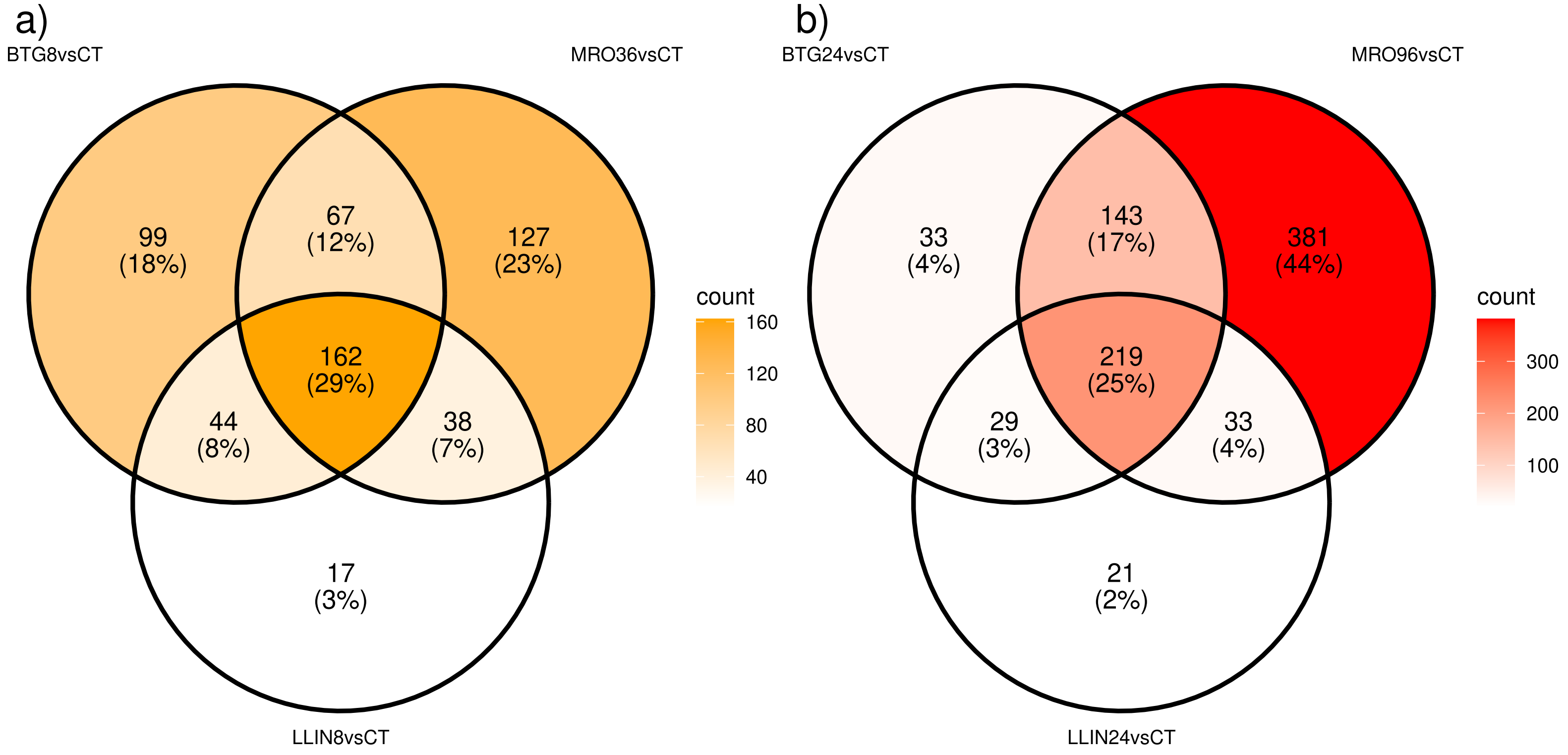

Supplementary Figure S5. Number and percentage of shared DEGs (*padj* < 0.05) among the three treatments. a) DEGs shared between the three treatments at the first time point; b) DEGs shared between the three treatments at the second time point. Different time points are shown in different colors, while the color gradient reflects the gene count (the darker the color, the higher the DEG count).
