# Supplementary material for: Behind the scenes of Popillia japonica integrated pest management: differentially expressed gene analysis following different control treatments
Source: BMC Genomics. 2025 Sep 1;26:788. doi: 10.1186/s12864-025-11949-4 (PMC12400702; doi:10.1186/s12864-025-11949-4)
Supplement: Supplementary file 1 — Supplementary Material 1. [file 12864_2025_11949_MOESM1_ESM.zip › FigS7.pdf]

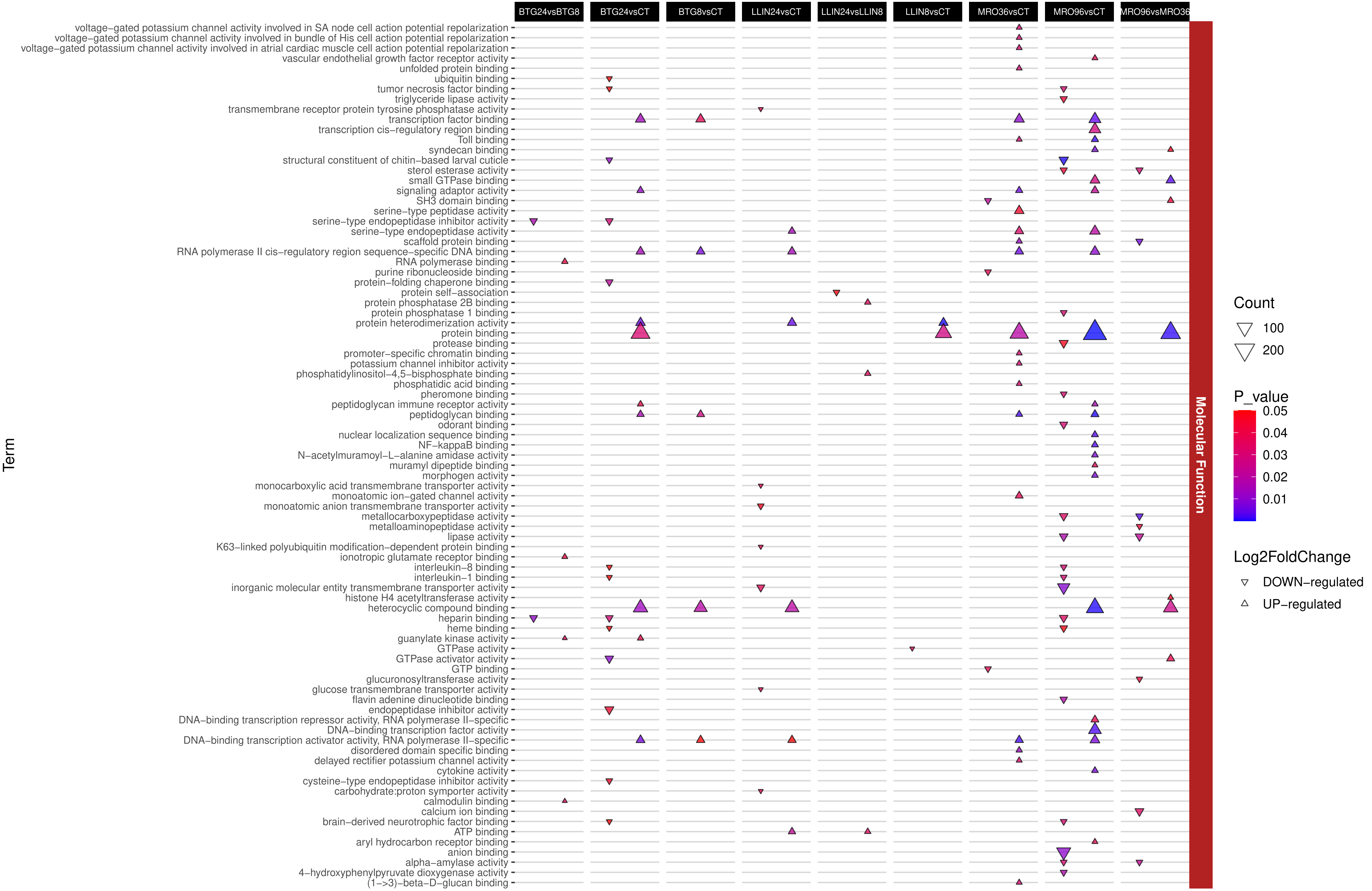

Supplementary Figure S7. Significant Molecular Function GO terms up or down regulated between different treatments and control. Shapes indicate an up- or down-regulation, their color indicates the *p-value* (< 0.05) and their size reflects the number of modulated GO terms.
